# Experimental Study on Grinding for Hole-Making of 2.5D C/SiC Composites Using Diamond Core Drills

**DOI:** 10.3390/ma19143007

**Published:** 2026-07-13

**Authors:** Bing Chen, Xuan Liu, Shiwei Sun, Rukai Liu, Weicai Quan, Jun Yi, Ye Guo

**Affiliations:** 1 Hunan Provincial Key Laboratory of High Efficiency and Precision Machining of Difficult-to-Cut Material, College of Mechanical Engineering, Hunan University of Science and Technology, Xiangtan 411201, China; 2 School of Intelligent Engineering, Xiangtan Institute of Technology, Xiangtan 411100, China

**Keywords:** 2.5D C/SiC composites, diamond core drills, grinding for hole-making, material removal mechanism, axial force, grinding temperature

## Abstract

2.5D C/SiC composites are characterized by high hardness and brittleness. These properties render the composites prone to fiber fracture, burr formation and matrix damage during grinding for hole-making. This study systematically investigates the material removal mechanism, tool wear behavior and machining quality evolution process during diamond core drill grinding for hole-making. Through experiments, the effects of grinding angle and grinding force and the thermal effects on material removal characteristics and hole wall machining quality were analyzed, and the stagewise characteristics of tool wear and the correlation between processing parameters and machining quality were clarified. The results indicate that the wear of diamond core drills undergoes a three-stage evolution: slight abrasive grain shedding at the initial stage, local damage and wear loss at the middle stage, and large-scale abrasive grain peeling followed by tool failure at the late stage. As wear aggravates, the machining axial force increases remarkably and the grinding temperature rises drastically. Hole wall defects gradually develop from minor initial fiber fracture into complex failure modes including burrs, fiber pull-out and matrix damage. In particular, the morphological degradation at the hole exit is the most severe. This study verifies that the optimization of process parameters and tool design can improve machining quality, and provides theoretical guidance and an experimental basis for the efficient and precise machining of high-performance composites.

## 1. Introduction

Performance requirements for high-performance structural materials in the aerospace sector continue to rise. Due to their excellent mechanical properties and high-temperature stability, 2.5D C/SiC composites are widely used in high-performance structural components such as aircraft engine parts, missile casings and thermal protection systems [1,2,3,4]. The 2.5-dimensional needle-punched structure of this material significantly enhances tensile strength and heat resistance. However, the marked differences in hardness and toughness between the carbon fibers and the ceramic matrix result in complex and non-uniform material removal characteristics [5,6,7]. Furthermore, the brittleness of the ceramic matrix tends to induce crack propagation, whilst the high strength of the carbon fibers increases the difficulty and uncertainty of machining operations [8,9]. These phenomena lead to defects such as cracks, burrs and delamination during machining [10,11], placing higher demands on the precision and efficiency of machining techniques.

Currently, existing drilling technologies for 2.5D C/SiC composites each have their limitations. Although conventional drilling features simple equipment and convenient operation, it suffers from poor hole wall quality, rapid tool wear, and low machining efficiency when processing high-hardness composites [12,13]. Laser drilling avoids mechanical contact between the tool and workpiece but is prone to thermal damage and hole wall carbonization induced by high temperatures, which degrades the service performance of the workpiece [14]. Ultrasonic drilling exhibits advantages in reducing grinding forces and improving hole wall quality, yet its high equipment cost and low machining efficiency make it difficult to satisfy industrial mass production requirements [15]. In contrast, diamond core drills integrate the material properties of high hardness and high wear resistance with the structural design advantages of hollow geometry. When machining high-hardness and high-brittleness materials, diamond core drills can effectively reduce heat accumulation and enhance hole wall quality [16,17]. Nevertheless, studies on the drilling of 2.5D C/SiC composites using diamond core drills remain scarce. The specific mechanisms by which grinding angles, tool wear, and thermal effects govern machining performance have not been fully elucidated, and systematic investigations into material removal mechanisms and thermomechanical behavior are urgently required.

In the existing research, scholars have attempted to improve the machining quality of composite materials by optimizing machining parameters and introducing cryogenic cooling technologies [18,19]. Although some progress has been made, there is still room for improvement in the machining adaptability of complex-structured ceramic matrix composites. To this end, this study selects 2.5D C/SiC composites as the workpiece and conducts a series of grinding for hole-making experiments using diamond core drills. The influence of grinding angle on the removal mechanisms of fiber and the matrix is systematically analyzed. The material removal characteristics at the hole entrance and exit during the grinding process are investigated. Combined with the evolution laws of axial force, grinding temperature and acoustic emission signals, the staged wear characteristics of the tool and the effects of machining parameters on hole wall quality and tool service life are revealed. This study aims to clarify the fundamental machining mechanism of 2.5D C/SiC composites machined by diamond core drills. It provides a theoretical basis for the engineering application of diamond core drills in hole-making for high-performance ceramic matrix composites and offers technical references for machining process optimization as well as the improvement of machining efficiency and quality.

## 2. Study on the Mechanism of Grinding for Hole-Making Using Diamond Core Drills

Grinding for hole-making using diamond core drills achieves material removal through abrasive interactions between the tool and workpiece. The grinding force, chip morphology, and hole wall quality are influenced by multiple factors such as grinding angle, machining position, spindle speed, feed rate and cooling condition. To reveal its material removal mechanism, this section conducts a systematic study from two core perspectives: the influence of grinding angle on fiber and matrix removal, and the material removal characteristics at the hole entrance and exit during the grinding for hole-making process.

### 2.1. Influence of Grinding Angle (θ) on the Material Removal Mechanism of Fibers and Matrix in C/SiC Composites

The grinding angle (θ) is defined as the angle between the grinding direction of abrasive particles and the axial direction of carbon fibers. Under different grinding angles, the force characteristics exerted by abrasive grains on fibers and the matrix vary, leading to distinct differences in material removal behavior and damage mechanisms.

**Figure 1:**
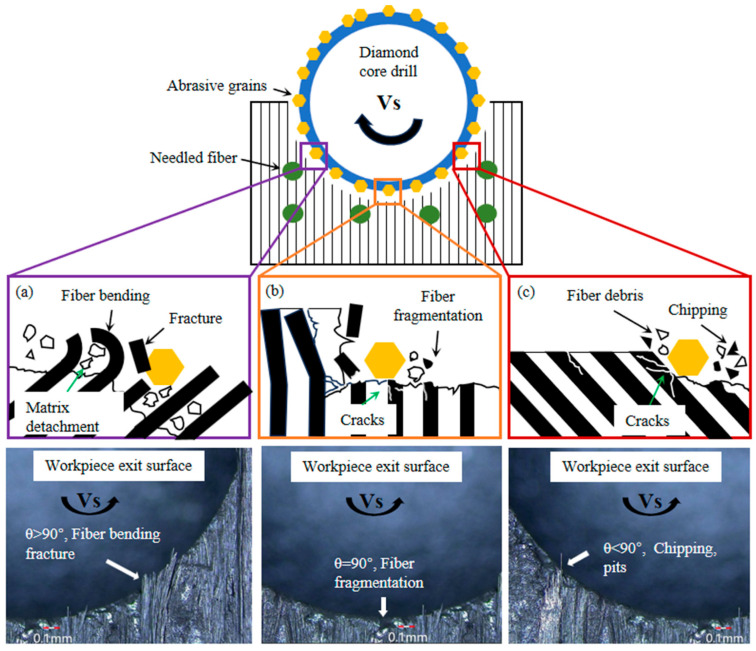
Diagrams of material removal mechanisms at different grinding angles: (**a**) θ > 90°; (**b**) θ = 90°; (**c**) 0° < θ < 90°.

(1) When θ > 90°, the force exerted by abrasive grains can be decomposed into a bending force along the fiber axis and a compressive stress perpendicular to the axis. Fibers undergo bending deformation under compressive stress, and the matrix gradually disintegrates. Due to the weak interfacial bonding between fibers and the matrix, microcracks easily initiate and propagate at the interface, resulting in matrix fragmentation and insufficient fiber support, eventually leading to fiber bending fracture, as shown in Figure 1a.

(2) When θ = 90°, the abrasive grains exert both compressive and shear forces on the fibers, causing them to undergo brittle fracture. This results in the formation of microscopic pits and microcracks at the fracture surface, which gradually propagate into fine, fragmented damage to the fibers, as shown in Figure 1b.

(3) When 0° < θ < 90°, the abrasive grains mainly exert compressive stress. This compressive stress aggravates fiber–matrix interfacial debonding and induces fiber chipping. The matrix shatters into fine residues under pressure and forms pits, as shown in Figure 1c.

In summary, when θ > 90°, the fibers are prone to bending and fracture; when θ = 90°, fiber fracture and fine fragmentation are the predominant forms of damage; and when 0° < θ < 90°, compressive stress is the primary force, and fiber chipping and fragmentation are more pronounced.

### 2.2. Study on Material Removal Mechanics at Hole Entrance and Exit During Core Drilling

**Figure 2:**
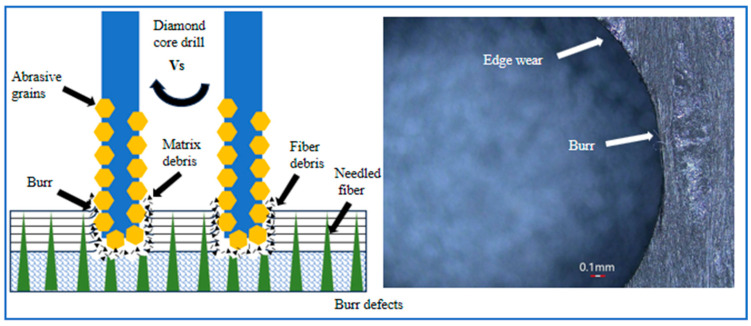
Material removal mechanism and defects at hole entrance.

**Figure 3:**
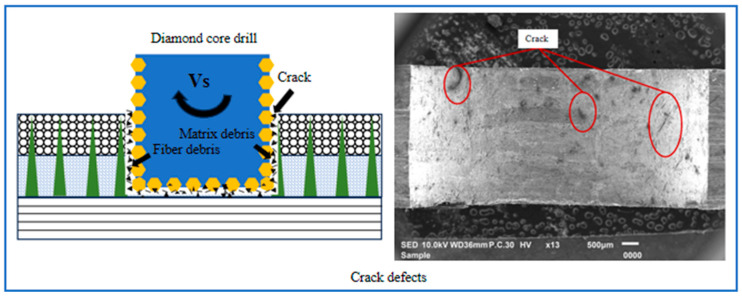
Material removal mechanism and defects at mid-stage.

**Figure 4:**
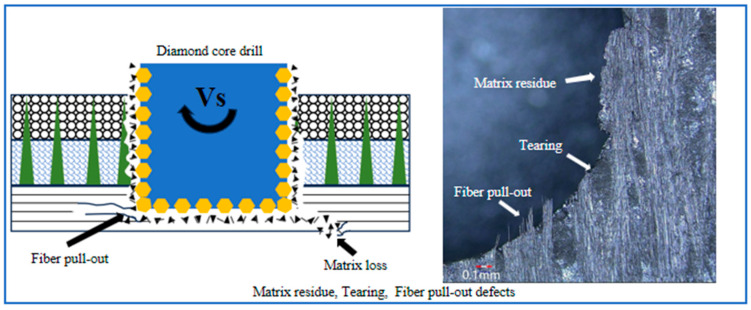
Material removal mechanism and defects at hole exit.

The grinding for hole-making process of 2.5D C/SiC composites using diamond core drills is governed by multiple factors, including the tool–workpiece contact condition and material support strength. The material removal mechanism exhibits distinct differences across the hole entrance, middle, and exit regions. In particular, the entrance and exit zones are recognized as high-risk locations for machining defects. The detailed characteristics are as follows:

(1) At the hole entrance, an instantaneous impact is generated at the initial contact between the tool and the workpiece surface. Under the action of axial force, the surface-layer carbon fibers are sheared by diamond abrasive grains. The material is mainly removed through brittle fracture, which results in entrance burrs and edge wear. The top-view morphology of the hole entrance is shown in Figure 2. Burr formation is mainly induced by brittle fracture of the carbon fibers. With further tool penetration into the workpiece, the grinding process gradually enters a stable grinding stage. The axial force then tends to stabilize, and the hole wall quality is correspondingly improved.

(2) Middle region: In the stable grinding stage, diamond abrasive grains perform continuous and uniform grinding on carbon fiber bundles and the SiC matrix, with material removal dominated by crack propagation. In this stage, the SiC matrix is uniformly removed, resulting in a smooth hole wall surface with few defects. The grinding force remains balanced, and the material removal process is steady. The front-view morphology of the hole wall surface is presented in Figure 3.

(3) At the hole exit, as the tool approaches the exit, insufficient support of the bottom-layer material leads to the axial force exceeding its load-bearing capacity, giving rise to defects including fiber bundle tearing, matrix residue, and fiber pull-out. The bottom-view morphology of the hole exit is shown in Figure 4. The needled fiber bundles are exposed at the exit, and the synergistic effects of grinding and friction further exacerbate material damage. In particular, during tool retraction, it is prone to inducing the further propagation of exit defects.

## 3. Experiments on Hole-Making Using Diamond Core Drills for 2.5D C/SiC Composites

**Figure 5:**
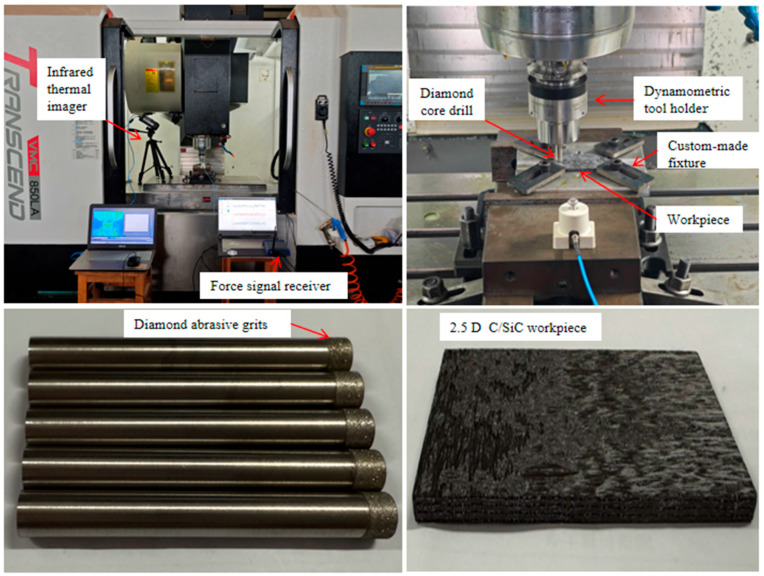
Experimental platform for hole-making of 2.5D C/SiC composites using diamond core drills.

The experimental setup for diamond core drilling of 2.5D C/SiC composites is illustrated in Figure 5. The workpiece used in the experiments was a 2.5D carbon fiber-reinforced ceramic matrix composite (C/SiC) produced by a commercial manufacturer, with dimensions of 60 mm × 35 mm × 4 mm. The diamond core drill employed in this experiment was fabricated by sintering mixed diamond abrasive grains and metal powders (cobalt, iron and nickel) under high temperature and high pressure, followed by compression molding. The drill has an outer diameter of 8 mm, a wall thickness of 0.5 mm, a total length of 60 mm, and an abrasive grain size of 180 mesh, with an effective drilling depth of 5 mm. A VMC850LA vertical CNC milling machine (Transcend, Changzhou, China) was employed for the experiments. The axial force, grinding temperature, and acoustic emission (AE) signals during machining were measured using a dynamometric tool holder (Spike^2^, Pro-micron, Kaufbeuren, Germany), an infrared thermal imager (FLIR ThermaCAM SC 3000, Teledyne FLIR, Wilsonville, OR, USA), and an AE system (SR150 AE sensor & SAEPA2 preamplifier, Physical Acoustics, Princeton Jct., NJ, USA; USB-6351 module, NI, Austin, TX, USA), respectively. The morphologies of the hole entrance and exit and the micro-morphology of the hole wall were characterized using an optical microscope (HM-FD600E, AOSVI, Shenzhen, China) and a scanning electron microscope (SEM, JSM-IT100, JEOL, Tokyo, Japan), respectively.

Based on previous studies by domestic and international researchers on grinding hole-making for carbon fiber composites [13,16,20,21], as well as preliminary experimental data obtained by our research group, the following experimental parameters were determined for continuous grinding for hole-making of 4 mm thick 2.5D C/SiC composites: spindle speed of 3000 r/min and feed rate of 8 mm/min. The specific experimental process parameters are presented in Table 1. The core performance parameters of the T700 carbon fibers and SiC ceramic matrix used in the experiments are listed in Table 2 and Table 3, respectively.

## 4. Results and Analysis

### 4.1. Axial Force

A dynamometric tool holder was used to record the axial force data during six consecutive grinding passes of 2.5D C/SiC composites using the diamond core drill. The results indicate that the single-pass machining process can be divided into three stages according to the variation characteristics of axial force: initial contact stage, stable grinding stage, and retraction stage. The core characteristics of each stage are as follows:

(1) Initial contact stage (0–2.5 s): When the core drill first contacts the workpiece surface, the axial force increases rapidly. This stage is affected by the uneven surface hardness, irregular shape of the workpiece, and feed rate, leading to local stress concentration and no large-scale material removal, which is a rapid rising stage of axial force.

(2) Stable grinding stage (2.5–26 s): The core drill is fully engaged with the workpiece, and material removal enters a stable stage. The axial force fluctuates slightly within the range of 15–37 N, and the fluctuations are mainly caused by the material inhomogeneity and changes in machining conditions.

(3) Retraction stage (26–30 s): After the completion of grinding, the core drill gradually detaches from the workpiece, the grinding area and load decrease rapidly, and the axial force drops sharply from 27 N to 0 N.

**Figure 6:**
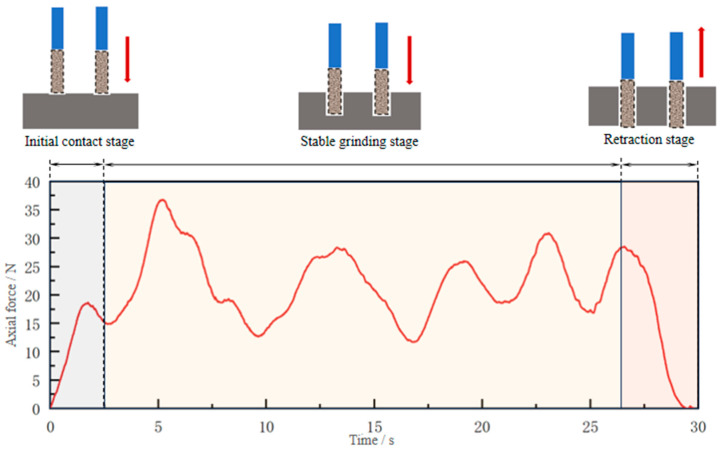
Axial force trend and machining stages during the first pass.

Taking the first pass as an example (Figure 6), the axial force rises rapidly to approximately 19 N in the initial contact stage, mainly due to the transition of the material from elastic deformation to plastic deformation. Local stress concentration and shear failure significantly increase the axial force. In the stable grinding stage, the axial force fluctuates periodically, which is affected by material removal and inhomogeneity. In the retraction stage, a rapid decrease in axial force was observed. This decrease was associated with the gradual detachment of the core drill from the workpiece.

**Figure 7:**
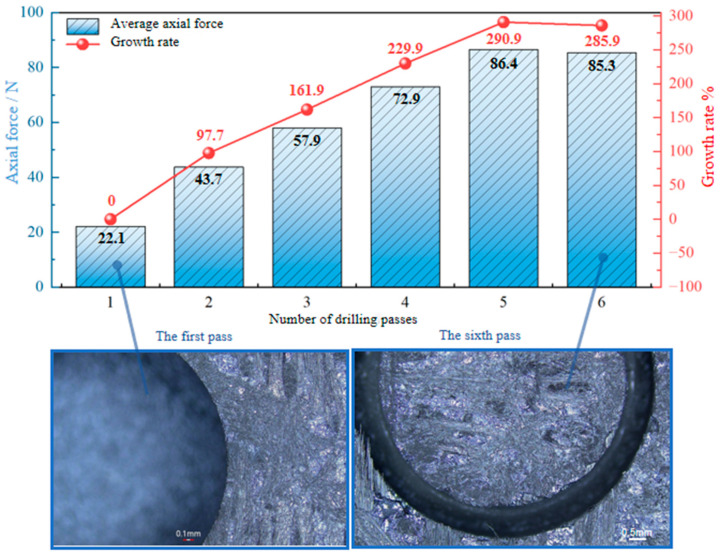
Average axial force and growth rate from the first to the sixth pass.

The stable grinding stage is crucial for analyzing the mechanical properties of the core drill and material response. It features a long time span and a large number of sampling points, which can reduce the impact of accidental fluctuations and improve statistical reliability. The average axial forces of the first to sixth passes are 22.1 N, 43.7 N, 57.9 N, 72.9 N, 86.4 N, and 85.3 N, respectively (Figure 7). The axial force increases from 22.1 N to 86.4 N in the first five passes, mainly due to the wear or shedding of diamond abrasive grains, which reduces the sharpness of the grinding edges. The thermal accumulation effect increases material softening, thermal expansion, and friction, thereby exacerbating grinding difficulty and load. Chip accumulation further increases the friction coefficient. The axial force decreases to 85.3 N in the sixth pass, which is an abnormal machining state. At this point, the tool has failed, with a large number of abrasive grains shedding, and the hole cannot be fully drilled; the main source of axial force is the conversion of grinding force into friction force.

The growth rates of the average axial force of the first to sixth passes relative to the first pass were calculated as 97.7%, 161.9%, 229.9%, 290.9%, and 285.9%, respectively. The growth rate increases gradually from the second to the fifth pass, especially showing a significant increase from the third to the fifth pass, indicating that tool wear continues to intensify and grinding resistance rises rapidly. The growth rate showed a slight decline at the sixth pass, which reflects the full failure of diamond abrasive grains and an essential degradation of the tool machining performance. The growth rate of the first five passes shows a linear change, indicating that tool wear and the material removal process are relatively uniform during this stage, and the machining conditions remain stable.

The variation in axial force directly determines machining stability and hole quality: when the axial force exceeds 70 N, the fiber bundles at the hole exit cannot be completely cut off, leading to severe fiber pull-out, increased burrs, and matrix damage. After tool failure, although the axial force decreases slightly, material removal is dominated by frictional action rather than effective grinding, which is likely to cause collapse and damage at the hole exit. Meanwhile, excessive axial force will further exacerbate fiber fracture and matrix fragmentation, thereby deteriorating the machining quality of the hole wall and reducing its service performance.

### 4.2. Grinding Temperature

**Figure 8:**
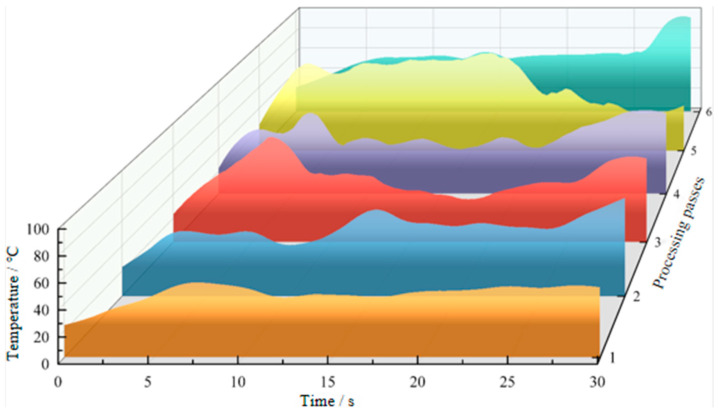
Grinding temperature waterfall chart from the first to the sixth pass.

During the grinding for hole-making process, temperature changes were measured by the FLIR ThermaCAM SC 3000 infrared thermal imager, with the material reflectivity set to 0.8 [22]. The results indicate that the grinding temperature is dominated by frictional heat. The temperature change in the tool synchronously presents dynamic characteristics corresponding to the three stages (initial contact, stable grinding, and retraction), which is consistent with the variation law of axial force. The 3D waterfall plot of temperature variation is shown in Figure 8, and the main characteristics of each stage are as follows:

(1) Initial contact stage (0–2.5 s): For the first pass, the temperature rises to 55.9 °C, showing high-efficiency grinding with low frictional heat generation. With the increase in grinding passes, tool wear intensifies and grinding efficiency decreases. The temperature of the subsequent passes gradually decreases, dropping to 42 °C in the sixth pass, which indicates a significant decline in the grinding ability of the tool.

(2) Stable grinding stage (2.5–26 s): The tool temperature is affected by both tool wear and heat dissipation capacity. The temperature stabilizes at 50 °C in the first pass, showing a good hole-making condition. Affected by uneven tool wear, the temperature rises to 78.2 °C in the second pass and reaches a peak of 88.5 °C in the third pass. At the initial stage of the fifth pass, the grinding temperature reached 85°C and then decreased to 42°C. This temperature variation suggested that the tool was approaching failure. During the sixth pass, the temperature remained at 55°C, while the heat dissipation capacity of the tool was severely weakened.

(3) Retraction stage (26–30 s): When the tool is detached from the workpiece, the temperature changes rapidly. During the first five passes, good heat dissipation performance was maintained by the tool. The grinding temperature decreased after retraction. During the sixth pass, a large amount of heat accumulated due to tool failure. As a result, the temperature increased to 94.6 °C after retraction, which was significantly higher than that measured during machining.

**Figure 9:**
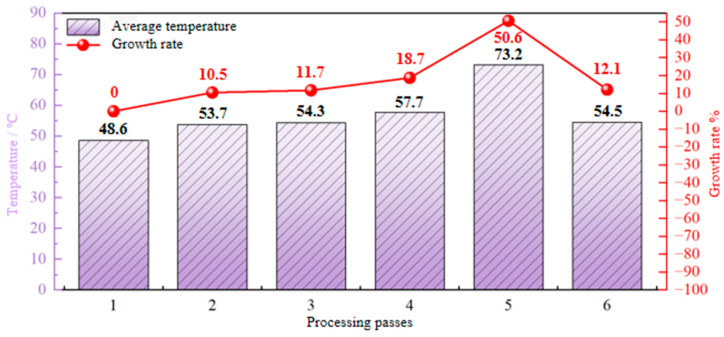
Average grinding temperature and growth rate.

The temperature variation during the stable grinding stage can better reflect the interaction law between the tool machining state and the material. In this stage, the tool temperature presents a phased upward trend from the first to the sixth pass, as illustrated in Figure 9. For the first to fourth passes, the temperature remains stable with a small fluctuation amplitude, indicating that the tool maintains high grinding efficiency and excellent heat dissipation performance during this period. For the fifth pass, the temperature surges to 73.2 °C, which is mainly attributed to the massive shedding of abrasive grains, leading to intensified accumulation of frictional heat during the grinding process. For the sixth pass, the temperature drops back to 54.5 °C, reflecting that the tool has completely failed and lost its grinding capability. The grinding process is converted into frictional action, resulting in a reduction in the generation of frictional heat.

The temperature growth rate presents an obvious phased pattern: the growth rates of the second to fourth passes are 10.5%, 11.7%, and 18.7%, respectively, showing a stable growth trend, which indicates that the tool is in the normal wear stage. The growth rate of the fifth pass surges to 50.6%, which is a significant signal that the tool is approaching failure. The growth rate of the sixth pass drops back to 12.1% because the grinding force decreases sharply, leading to a reduction in the generation of frictional heat. Overall, the machining process can be divided into three stages according to temperature variation. The first to fourth passes are defined as the initial stage, with stable temperature and favorable machining performance. The fifth pass corresponds to the accelerated wear stage, in which the temperature rises abruptly and frictional heat accumulates significantly. The sixth pass is regarded as the severe failure stage, where the grinding capability decreases markedly and the heat dissipation capacity is severely weakened.

Experiments show that the tool temperature generally increases with the number of grinding passes, affected by tool wear, material properties, and heat dissipation capacity. The increase in grinding temperature not only directly affects tool service life but also has a significant negative impact on hole wall machining quality. High temperatures reduce the interfacial bonding force between carbon fibers and the SiC matrix, making fiber pull-out more significant, while also exacerbating the initiation and propagation of cracks on the hole wall. Due to the weak material support force at the hole exit, it is more severely affected by high temperatures, which are prone to causing fiber tearing and further deterioration of exit burr defects.

### 4.3. Hole Entrance and Exit Morphologies

During the grinding for hole-making of 2.5D C/SiC composites, defects such as burrs, tearing, and delamination are likely to occur at the hole entrance and exit, affecting machining quality and workpiece performance. To address this, the morphologies of the hole entrance and exit were analyzed in this study.

**Figure 10:**
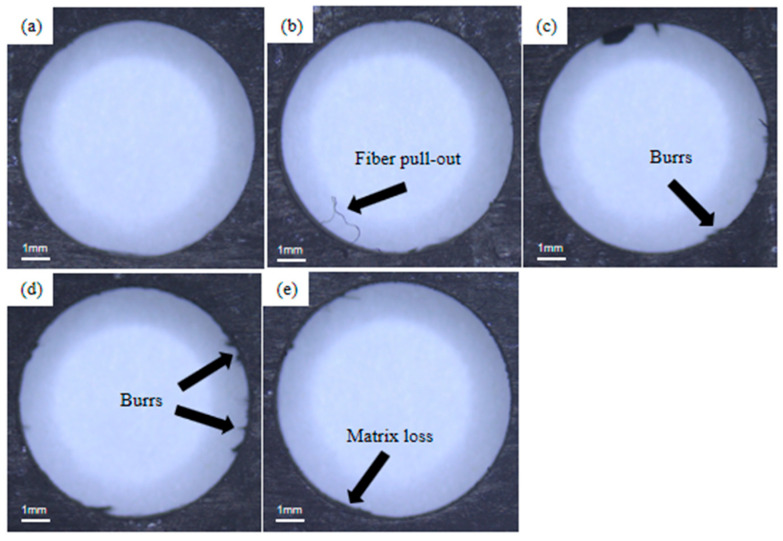
Inlet morphology of drilled holes: (**a**) first hole; (**b**) second hole; (**c**) third hole; (**d**) fourth hole; (**e**) fifth hole.

As shown in Figure 10, when drilling the first hole, the tool retains a well-preserved surface morphology with sharp abrasive grits, which facilitates stable cutting. No entrance defects are detected (Figure 10a), and the hole entrance exhibits superior machined quality. As drilling proceeds to the second hole, slight fiber pull-out initiates at the entrance (Figure 10b). Concurrent incipient wear of abrasive grits elevates grinding resistance and localized contact stress. For holes 3 to 5, progressive abrasive wear develops on the tool surface. Despite the relatively intact overall entrance contour, minor fiber pull-out and matrix loss sporadically emerge (Figure 10c–e), and the entrance quality remains generally acceptable. At the sixth drilling pass, severe wear and blunting of abrasive grits occur, which renders the tool unable to effectively remove fiber and matrix materials, ultimately leading to drilling failure.

**Figure 11:**
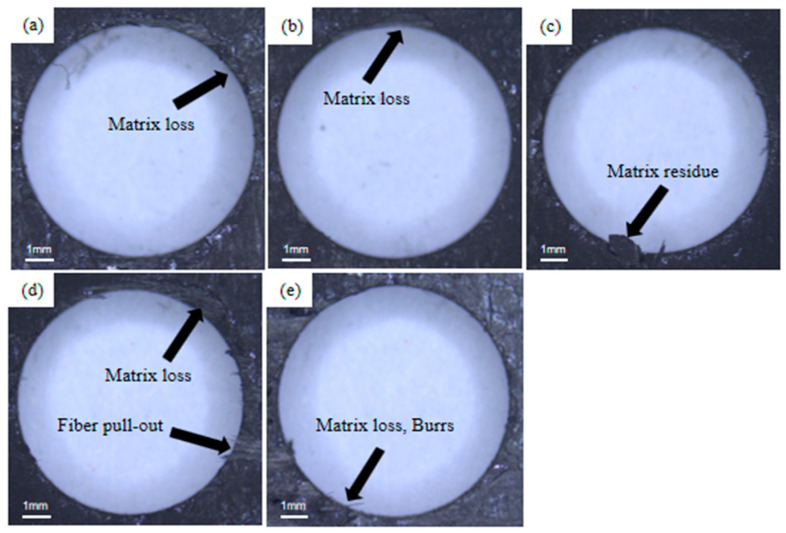
Outlet morphology of drilled holes: (**a**) first hole; (**b**) second hole; (**c**) third hole; (**d**) fourth hole; (**e**) fifth hole.

Compared with the hole entrance, the hole exit morphology is more susceptible to tool wear. The exit surface of the first hole demonstrates excellent integrity, with only marginal matrix loss observable. Sharp abrasive grits enable clean fiber cutting without the formation of additional machining defects (Figure 11a). For the second hole, the extent of matrix loss expands noticeably. Degradation in grit sharpness aggravates interfacial friction and thermal loading, ultimately inducing severe material damage at the hole exit (Figure 11b). Slight matrix residue accumulates on the exit surface of the third hole, whereas distinct burrs and large-scale material defects are absent (Figure 11c). Subsequent abrasive grit shedding occurs during the machining of the fourth and fifth holes, giving rise to prominent matrix loss, fiber pull-out and edge burrs on the hole exit surfaces. This wear evolution reduces material removal efficiency and leads to pronounced deterioration of exit surface quality (Figure 11d,e).

**Figure 12:**
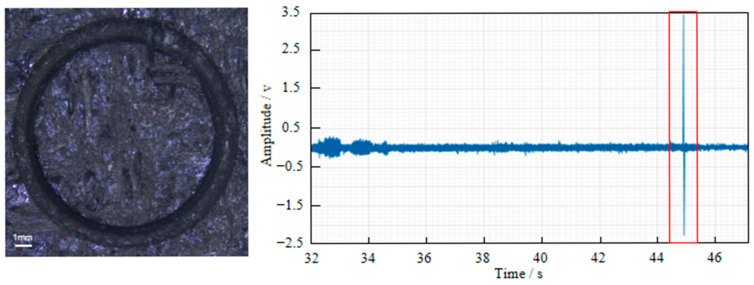
Morphology and acoustic emission signals of the sixth hole.

**Figure 13:**
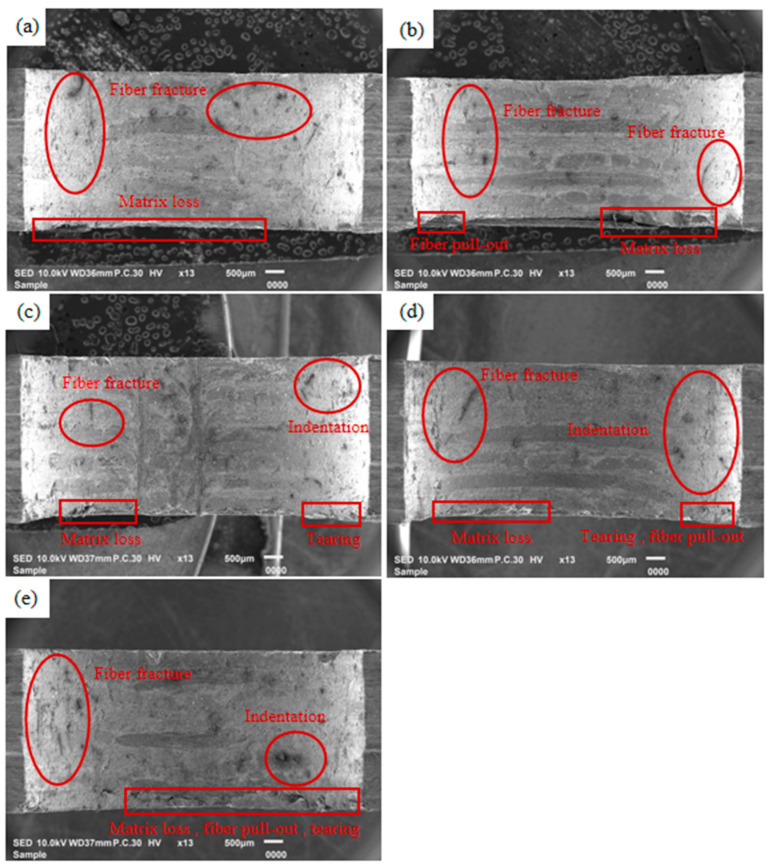
Overall wall morphology of drilled holes: (**a**) first pass; (**b**) second pass; (**c**) third pass; (**d**) fourth pass; (**e**) fifth pass.

The sixth hole was not fully drilled due to complete tool failure, leaving only a groove on the workpiece surface (Figure 12). At this point, the diamond abrasive grains of the tool have completely disappeared, the grinding function is lost, and machining is converted into pure friction and extrusion. The groove morphology indicates that chip accumulation and tool smoothing lead to the inability to remove material. During the sixth drilling pass, a low AE signal amplitude and slight fluctuations were observed. This signal response indicated a reduction in grinding capability. Near the final stage of the sixth pass, abnormal fluctuations in the AE signal were observed at 44–46 s, as shown in Figure 13. These fluctuations may be attributed to enhanced sliding friction, local deformation, and crack propagation under high temperature and high load.

### 4.4. Hole Wall Micro-Morphology

The micro-morphology of the hole wall during the grinding of 2.5D C/SiC composites using diamond core drills is affected by both the structural characteristics of the material itself and the degree of tool wear. Due to the complexity of the carbon fiber bundle and needled fiber structure, machining defects present obvious regional distribution characteristics and gradually worsen with tool wear.

(1) Overall wall morphology of drilled holes

As shown in Figure 13, an analysis of the hole wall morphologies from the first to the fifth passes reveals that defects are concentrated in the two side regions of the hole wall, especially in the regions with large grinding angles, while there are few defects in the middle region. The hole wall morphology of the first pass is uniform. Fiber fracture is obvious on both sides, while the central region presents a smooth surface (Figure 13a). For the second pass, defects on both sides increase, and fiber fracture and pull-out become more severe, whereas the central region remains relatively flat (Figure 13b). In the third pass, defects on both sides increase significantly, with surface indentation and matrix loss, and the flatness of the central region is still well maintained (Figure 13c). In the fourth pass, fiber tearing and pull-out on both sides are further aggravated, especially at the outlet region. The surface quality of the central region is still superior to that of both sides (Figure 13d). The fifth pass exhibits the most severe defects, which are concentrated on both sides in the form of fiber pull-out, tearing and matrix loss, especially at the hole outlet (Figure 13e). Although certain defects also exist in the central region, it remains relatively smooth in the fifth pass.

(2) Local morphology of fibers

**Figure 14:**
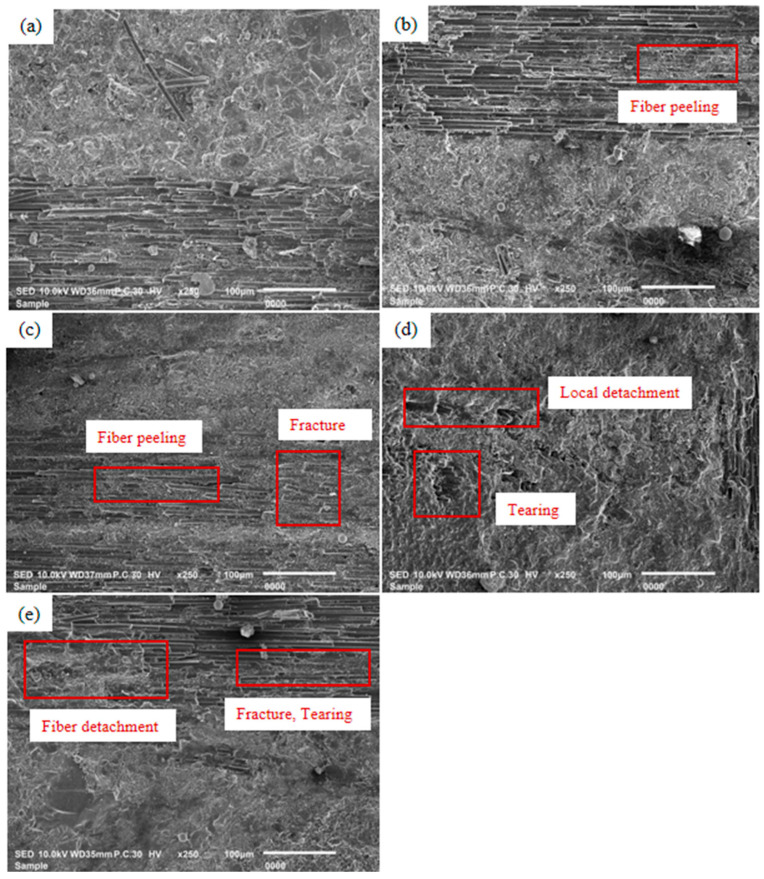
Local morphology of fibers: (**a**) first pass; (**b**) second pass; (**c**) third pass; (**d**) fourth pass; (**e**) fifth pass.

In the continuous grinding-based hole-making process of 2.5D C/SiC composites using diamond core drills, the continuous aggravation of tool wear gradually changes the micro-morphology of fibers on the hole wall from regular to disordered. The layered characteristics of fiber bundles are weakened, and fiber fracture and abrasive damage are continuously aggravated, as shown in Figure 14.

In the first pass, the cutting edge of the tool remains sharp with low grinding force. The fibers on the hole wall are neatly arranged, featuring flat fracture surfaces and distinct layered structures. The interfacial bonding between fibers is tight, without obvious defects such as fiber tearing and detachment (Figure 14a). In the second pass, slight tool wear leads to a small increase in grinding force. Slight deformation occurs on the fibers of the hole wall, the roughness of the fiber fracture surfaces increases, and the layered characteristics are weakened. Fiber fracture is still relatively uniform, and matrix peeling defects start to appear on the surface (Figure 14b). In the third pass, tool wear is significantly intensified and the grinding force rises further. The fiber fracture surfaces become rough and irregular with widened and deepened fracture zones. The local interfacial bonding strength of fibers decreases, matrix peeling occurs, and the surface quality of the hole wall deteriorates (Figure 14c). In the fourth pass, tool wear enters a rapid development stage and the grinding force keeps rising. The boundaries of fiber fracture become blurred, and the layered characteristics almost disappear. A large number of microcracks generate on the hole wall. Under high grinding loads, partial fiber bundles shift and detach locally, resulting in a sharp increase in machining defects (Figure 14d). In the fifth pass, the tool approaches failure, accompanied by a sharp surge in grinding force and grinding temperature. The layered structure of fibers completely disappears, and the fracture zones show severe roughness. The hole wall is covered with dense cracks and various damages. Local fiber bundles peel off in large areas or even separate entirely from the hole wall, causing severe damage to the hole wall (Figure 14e).

### 4.5. Analysis of Tool Wear

**Figure 15:**
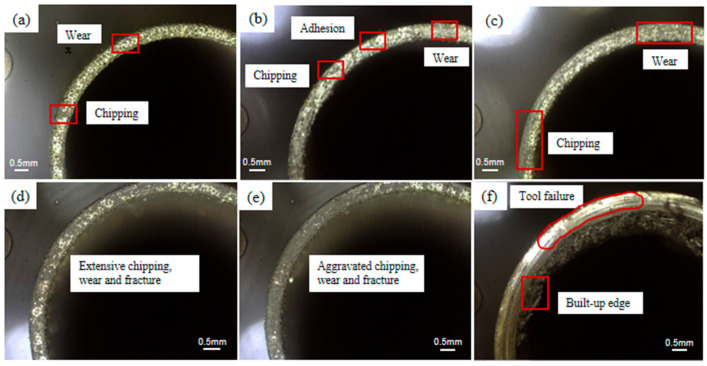
Morphologies of the tool after grinding: (**a**) first pass; (**b**) second pass; (**c**) third pass; (**d**) fourth pass; (**e**) fifth pass; (**f**) sixth pass.

The intrinsic ultrahigh wear resistance of 2.5D C/SiC composites drastically accelerates the wear rate of diamond tools, which directly degrades the surface quality of machined holes and shortens tool service life. After drilling each hole, the end face of the tool was characterized and photographed using electron microscopy. Two dominant wear modes were identified: diamond grain fracture and grain chipping. The entire evolution of tool wear is presented in Figure 15.

After the first grinding pass, only mild wear and minor localized chipping of diamond grains were observed, and the cutting edge remained intact (Figure 15a). Carbon fibers feature high hardness and inherent brittleness; transient impact loads generated during the initial cutting cycle are the primary cause of minor grain spalling. During the second pass, tool wear was significantly aggravated, and a prominent built-up edge (BUE) formed on the cutting edge (Figure 15b). High temperatures induced by dry cutting strengthen the interfacial bonding strength between the workpiece and tool, leading carbonaceous workpiece debris to firmly adhere to the tool surface and accelerate material loss of the diamond tool. During the third pass, chipped regions continuously expanded, reducing the tool’s effective cutting area. The drastically elevated localized contact stress further intensified the wear loss of the residual intact diamond grains (Figure 15c). After the fourth pass, the tool exhibited multiple severe damage modes, including extensive abrasive wear, large-scale grain chipping and substrate fracture (Figure 15d). Local grain spalling directly deteriorated the cutting sharpness, increased grinding resistance and induced massive heat accumulation. Under the coupled action of high temperature and cyclic mechanical stress, microcracks nucleated on the tool surface and caused severe structural damage in local regions. Tool degradation worsened continuously in the fifth pass, accompanied by large-area chipping of diamond grains. The voids left by detached grains were filled with carbon fiber matrix debris (Figure 15e). Rapid depletion of the wear-resistant diamond layer raised interfacial friction, and the tool substrate could no longer withstand cyclic impact loads from the workpiece, generating extensive fractured zones. By the sixth pass, catastrophic tool failure took place. The cutting edge turned smooth, nearly all diamond grains detached from the substrate, and massive chips accumulated within the tool cavity (Figure 15f). Trapped chips clogged chip evacuation paths and aggravated cutting heat accumulation, ultimately rendering the tool fully incapable of material removal.

Tool wear behavior modulates not only axial grinding forces and cutting temperature but also the amplitude of acoustic emission (AE) signals. To extract characteristic AE signatures corresponding to distinct wear stages, signals within a 23.5 s steady grinding window (10 s before and after the midpoint of stable material removal) were analyzed.

**Figure 16:**
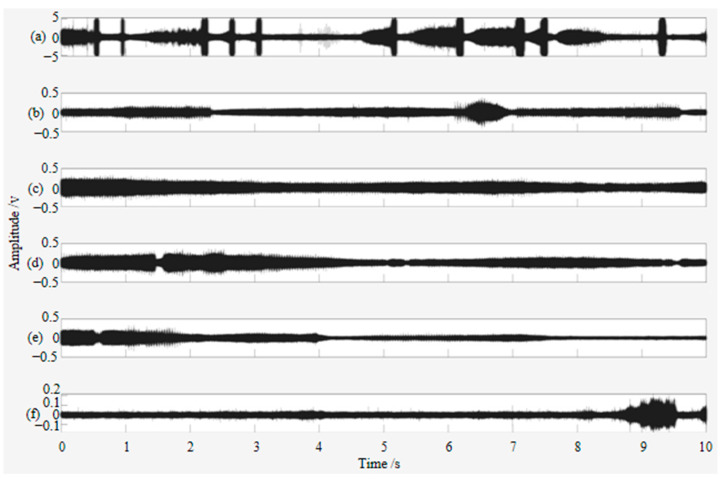
Acoustic emission signals recorded during machining operations: (**a**) first pass; (**b**) second pass; (**c**) third pass; (**d**) fourth pass; (**e**) fifth pass; (**f**) sixth pass.

AE signals collected during the first pass exhibited high amplitude with steady fluctuations (Figure 16a), which reflected favorable cutting performance and stable material removal. At this stage, only mild wear and minor localized grain chipping were observed, imposing negligible disturbance on machining stability. In the second pass, the AE amplitude decreased slightly, accompanied by drastically intensified signal fluctuations (Figure 16b), which corresponded to aggravated tool wear and elevated axial grinding forces. Electron microscopy observations verified aggravated grain chipping and built-up edge formation on the cutting edge. The enhanced interfacial friction disrupted stable grinding behavior. The AE amplitude rebounded in the third pass with smoothed signal fluctuations (Figure 16c). This phenomenon originated from expanded wear zones, reduced effective cutting area and drastically elevated localized contact stress. AE signals acquired in the fourth pass displayed abrupt irregular fluctuations, where the amplitude plummeted before recovering (Figure 16d). This unstable signal characteristic stemmed from large-scale grain chipping and substrate fracture. Vibration noise induced by crack propagation and fiber fragmentation further magnified signal volatility. The AE waveform recorded in the fifth pass presented highly complex chaotic fluctuations (Figure 16e), indicating that the tool had entered the accelerated wear regime. At this stage, grinding resistance and interfacial friction reached their peak values; nearly all diamond grains detached, the axial force surged drastically, and the tool suffered irreversible wear failure. In the sixth pass, the overall AE amplitude declined and waveform fluctuations became gentle (Figure 16f), revealing that the material removal mechanism had transitioned from cutting-dominated to friction-and-extrusion-governed. The tool experienced complete catastrophic failure, with smooth cutting edges stripped of diamond grains and covered by massive trapped chips. A transient rebound in signal amplitude observed in the late machining phase is presumed to be associated with stress accumulation caused by incomplete hole penetration.

Systematic analysis of diamond tool wear evolution during 2.5D C/SiC drilling fully clarifies the underlying wear mechanisms and their regulatory impacts on machining performance. The experimental results indicate that repeated machining passes induce progressive diamond grain chipping and continuous loss of cutting sharpness, which directly increases grinding resistance and degrades material removal capability. Mild tool wear and stable grinding can be maintained at the initial machining stage. As the machining process continues, the dominant damage mode transitions from minor localized grain chipping to large-scale grain spalling, substrate fracture and extensive built-up edge accumulation.

## 5. Conclusions

Through studying the performance of diamond core drills in the grinding for hole-making of 2.5D C/SiC composites, the interaction mechanism between the tool and material in different machining stages and its influence on machining quality were analyzed. The main conclusions are as follows:

(1) The grinding angle affects the material removal mode of 2.5D C/SiC composites. When θ > 90°, the fibers are prone to bending and fracture. When θ = 90°, fiber fracture and fine fragmentation are the predominant forms of damage. When 0° < θ < 90°, compressive stress is the primary force, and fiber chipping and fragmentation are more pronounced.

(2) The axial force climbs from 22.1 N to 86.4 N within the first five passes before undergoing a slight drop in the sixth pass. When the axial force exceeds 70 N, the defects at the hole exit deteriorate sharply, with tool wear and enhanced friction being the core inducing factors.

(3) The grinding temperature changes in phases with tool wear. The temperature of the first five passes is stable first and then surges to 73.2 °C, and the temperature of the sixth pass drops back but reaches 94.6 °C in the retraction stage. High temperature reduces the fiber–matrix bonding force, exacerbates hole wall defects, and has a particularly significant impact on the exit region.

(4) The machining quality gradually deteriorates with tool wear. The defects at the hole entrance and exit evolve from single-type to composite-type. The exit defects are much more serious than the entrance defects, and the hole wall defects are concentrated in the two side regions.

(5) Tool wear exhibits an evolutionary characteristic of slight abrasive grain shedding at the initial stage, local breakage and attrition at the middle stage, and large-scale grain peeling as well as tool failure at the later stage. The aggravation of tool wear induces an increase in axial force and grinding temperature, which acts as the core factor governing machining performance and hole-making quality. The optimization of machining parameters and tool structure can reduce the tool wear rate. It can also improve the machining quality and efficiency of grinding hole-making of 2.5D C/SiC composites. These results provide a technical basis for the efficient and precise machining of high-performance ceramic matrix composites.

## Figures and Tables

**Table 1:** Experimental process parameters.

Processing Method	Spindle Speed n/(r/min)	Feed Rate Vf/(μm/rev)
Dry machining	3000	2.67

**Table 2:** Properties of carbon fiber T700.

Material	Tensile Strength (MPa)	Tensile Modulus (GPa)	Density (g/cm^3^)	Thermal Expansion Coefficient (10^−6^·K)	Thermal Conductivity [W/(m·K)]	Fiber Diameter (μm)
T700	4900	230	1.8	−0.4 × 10^−6^	10	7

**Table 3:** Properties of SiC ceramic matrix.

Material	Tensile Strength (MPa)	Elastic Modulus (GPa)	Density (g/cm^3^)	Thermal Expansion Coefficient (10^−6^·K)	Thermal Conductivity [W/(m·K)]	Fracture Toughness (MPa·m1/2)
SiC	200	400	3.1	4.5 × 10^−6^	120	4

## Data Availability

The original contributions presented in this study are included in the article. Further inquiries can be directed to the corresponding author.

## References

[1] Liu C., Hou Z.X., Zhang J.T., Yang T. (2025). Toward understanding the damage behavior during orthogonal grinding of 2.5D C/SiC composite based on multi-scale modeling and high-speed photography. Proc. Inst. Mech. Eng. B J. Eng. Manuf..

[2] Zhang L.F., Wang S., Li Z., Qiao W.L., Wang Y., Wang T. (2019). Influence factors on grinding force in surface grinding of unidirectional C/SiC composites. Appl. Compos. Mater..

[3] Li J.C., Chen G.J., Xu J.K., Yu H.D. (2024). Study on material damage mechanism and surface quality of C/SiC composites by laser-ultrasonic hybrid micromachining. J. Mech. Eng..

[4] Jiao H.W., Chen B., Zuo B. (2021). Research progress in preparation and processing technology of C/SiC composites. J. Aeronaut. Mater..

[5] Cao C., Song Q.H., Fu H., Ji H.S., Liu Z.Q., Jiang L.P. (2023). Fiber orientation effects on grinding characteristics and removal mechanism of 2.5D Cf/SiC composites. Chin. J. Aeronaut..

[6] Li Y.F., Wen Q., Gong Y.D., Tang B.J. (2024). Experimental Study on Micro-scale Grinding of 2.5D Cf/SiC Composites. J. Northeast. Univ. (Nat. Sci.).

[7] Mao J.Z., Zhou J.P., Wang B.B. (2025). Study of ablation behavior on 2.5-dimensional C/SiC composites processed via short electric arc milling with various parameters. Proc. Inst. Mech. Eng. Part C J. Mech. Eng. Sci..

[8] Guo Y., Chen B., Zeng H.Y., Qing G.Y., Guo B. (2024). Research on wear state identification of ordered grinding wheel for C/SiC composites based on DBO-ELM. Wear.

[9] Yu P., Yu Z.Y., Wang L.Z., Gao Y.C., Li Q., Li Y.Q. (2025). Research on machining characteristics of C/SiC composite material by EDM. Micromachines.

[10] Zhang B.Y., Sui T.Y., Lin B., Zheng W., Li S.P., Sheng F., Huang Y., Feng Y.Q. (2022). Drilling process of Cf/SiC ceramic matrix composites: Grinding force modeling, machining quality and PCD tool wear analysis. J. Mater. Process. Technol..

[11] Zhou Y.G., Jia S.Q., Lu Y.Z., Liu J., Ma L.J., Li M., Li D.Z., Yin G.Q. (2024). Study on ultrasonic elliptical vibration-assisted grinding mechanism and surface quality of C/SiC composite material. Diam. Relat. Mater..

[12] Zhang C., Li H., Zhang X.X., An Q.L., Chen L. (2025). Parameter optimization, machining quality, removal mechanism, and tool wear in ultrasonic-vibration-assisted micro-hole drilling process of Cf/SiC ceramic matrix composites. Ceram. Int..

[13] He J.Y., Qian N., Su H.H., Fu Y.C., Ding W.F., Xu J.H. (2023). Wear behavior and machining quality of novel high-sharp brazed diamond abrasive core drills during drilling SiCf/SiC composite micro-holes. Int. J. Adv. Manuf. Technol..

[14] Liu C., Zhang X.Z., Gao L., Jiang X.G., Wang X.D., Yang T. (2021). Study on damage characteristics and ablation mechanism in fiber laser trepan drilling of 2.5D Cf/SiC composites. Int. J. Adv. Manuf. Technol..

[15] Liu Y., Liu Z.B., Wang X.B., Huang T. (2021). Experimental study on grinding force and surface quality in ultrasonic vibration-assisted milling of C/SiC composites. Int. J. Adv. Manuf. Technol..

[16] He W.B., Xu J.H., Nian Z.W., Yang Y.F., Zhao G.L. (2024). Investigation on high-precision drilling of Cf/SiC composites with brazed diamond core-drill. Mater. Today Commun..

[17] Abdullah A.B., Sapuan S.M. (2019). Hole-Making and Drilling Technology for Composites: Advantages, Limitations and Potential.

[18] Kang R.K., Ma H.N., Wang Z.W., Dong Z.G., Bao Y. (2023). Effect of tool wear on machining quality in milling Cf/SiC composites with PCD tool. J. Manuf. Process..

[19] Liu R.K., Chen B., Guo Y., Li S.S., Dai J.C. (2026). A review of cooling techniques for machining carbon fiber-reinforced composites. Int. J. Adv. Manuf. Technol..

[20] Irazu E., Alonso U., Izquierdo B., Godino L. (2024). Grinding of C/SiC ceramic matrix composites: Influence of grinding parameters on tool wear. Wear.

[21] Chen J., An Q.L., Ming W.W., Chen M. (2019). Hole exit quality and machined surface integrity of 2D Cf/SiC composites drilled by PCD tools. J. Eur. Ceram. Soc..

[22] Guo Y., Chen B., Fu X.J., Sun S.W. (2024). Experimental study on grinding 2.5D C/SiC composites by electroplated grinding wheel with ordered abrasive clusters. Diam. Relat. Mater..

